# Monomer Release from Dental Resins: The Current Status on Study Setup, Detection and Quantification for In Vitro Testing

**DOI:** 10.3390/polym14091790

**Published:** 2022-04-27

**Authors:** Tristan Hampe, Andreas Wiessner, Holm Frauendorf, Mohammad Alhussein, Petr Karlovsky, Ralf Bürgers, Sebastian Krohn

**Affiliations:** 1 Department of Prosthodontics, University Medical Center Göttingen, 37075 Göttingen, Germany; andreas.wiessner@med.uni-goettingen.de (A.W.); ralf.buergers@med.uni-goettingen.de (R.B.); sebastian.krohn@med.uni-goettingen.de (S.K.); 2 Institute for Organic and Biomolecular Chemistry, University of Göttingen, 37077 Göttingen, Germany; holm.frauendorf@chemie.uni-goettingen.de; 3 Molecular Phytopathology and Mycotoxin Research, University of Göttingen, 37077 Göttingen, Germany; mohammad.alhussein@uni-goettingen.de (M.A.); pkarlov@gwdg.de (P.K.)

**Keywords:** materials testing, resin based dental materials, biocompatibility, monomer, bisphenol A, elution, leaching

## Abstract

Improvements in mechanical properties and a shift of focus towards esthetic dentistry led to the application of dental resins in various areas of dentistry. However, dental resins are not inert in the oral environment and may release monomers and other substances such as Bisphenol-A (BPA) due to incomplete polymerization and intraoral degradation. Current research shows that various monomers present cytotoxic, genotoxic, proinflammatory, and even mutagenic effects. Of these eluting substances, the elution of BPA in the oral environment is of particular interest due to its role as an endocrine disruptor. For this reason, the release of residual monomers and especially BPA from dental resins has been a cause for public concern. The assessment of patient exposure and potential health risks of dental monomers require a reliable experimental and analytical setup. However, the heterogeneous study design applied in current research hinders biocompatibility testing by impeding comparative analysis of different studies and transfer to the clinical situation. Therefore, this review aims to provide information on each step of a robust experimental and analytical in vitro setup that allows the collection of clinically relevant data and future meta-analytical evaluations.

## 1. Introduction

Direct dental restorations of posterior teeth have been carried out with various materials, such as dental amalgam or composite resin [1]. Despite the successful application due to the high functional durability of dental amalgam for more than 150 years with a small number of reports on adverse effects [2,3], amalgam is being phased out due to the rise of safety concerns and the restriction of amalgam in some regions of the world [4,5,6]. Improvements in mechanical properties and a shift of focus towards esthetic dentistry led to the application of dental resins in various areas of dentistry [7,8], e.g., as restorative composites, bonding agents, resin-based cements, fissure, and root canal sealers as well as temporary crowns and bridges [9,10,11,12]. The specific monomer composition of dental resins is tailored to the particular area of application [13] and generally consists of one or more monomers, mostly bisphenol A diglycidyl methacrylate (Bis-GMA) and/or urethane dimethacrylate (UDMA) in addition to co-monomers, which are predominantly triethylene glycol dimethacrylate (TEGDMA) and 2-hydroxylethyl methacrylate (HEMA) [14]. Typical Bis-GMA/TEGDMA mixtures have a ratio between 60 and 80 wt.% Bis-GMA and 20 and 40 wt.% TEGDMA [15,16,17]. In combination with UDMA, less TEGDMA is required and most ratios between UDMA and Bis-GMA are possible, even complete replacement [15,17].

Bis-GMA is either the reaction product of bisphenol A (BPA) and glycidyl methacrylate or methacrylic acid and diglycidyl ether of bisphenol A (BADGE or DGEBA) [18]. Due to its low shrinkage, good mechanical properties, and excellent adhesion to enamel [19], Bis-GMA is the base monomer of most dental resins [20]. The central core of Bis-GMA is formed by a phenyl ring and two pendant hydroxyl groups, which are responsible for its extremely high viscosity and low mobility [21].

UDMA is the reaction product of 2-hydroxyethyl methacrylate and 2, 4, 4- trimethyl-hexamethylenediisocyanate and was developed by Foster and Walter in 1974 [22]. Instead of a phenol ring UDMA has an aliphatic urethane chain, which leads to higher flexibility and lower viscosity and results in higher mobility and a greater degree of conversion [23,24]. Due to these advantageous properties and health concerns regarding the release of bisphenol A (BPA) and its derivatives, more and more manufacturers substitute Bis-GMA with UDMA and introduced BPA-free composites to avoid the release of BPA and its derivatives [25,26,27].

Bis-GMA and UDMA are combined with a low-viscosity monomer such as TEGDMA, which improves the degree of conversion, filler loadings, and clinical handling [28]. TEGDMA is the reaction product of two molecules of methacrylic acid and triethylene glycol [18]. Its weaker polar hydrogen bonds lead to greater flexibility and its small size and its high number of double bonds increase conversion [29,30]. TEGDMA is only used as a co-monomer because its hydrophilicity amplifies undesirable properties like water sorption and polymerization shrinkage [31].

HEMA is a common co-monomer in dental adhesives and is characterized by its small dimensions and polar properties [28]. The major advantage of HEMA, especially in dental adhesives, is its ability to improve the miscibility between hydrophilic and hydrophobic monomers and thus dentine adhesion [32].

However, as of today, studies on the short-term release of compounds from the polymer network of composite resins are poorly comparable, studies on the long-term release are still rare, and degradation products are often not measured [33,34,35,36]. Due to its role as an endocrine disrupter of different metabolic pathways even in low concentrations [37], the release of bisphenol A (BPA) is of great interest in recent literature [38]. BPA interacts with the estrogen receptor and mimics the behavior of the natural hormone estradiol [39,40,41]. Furthermore, it is known that BPA exhibits potential cancerogenic, embryotoxic, and metabolic effects [40,42,43]. However, pure BPA is not being used as a monomer in dentistry, but rather as a reagent for the synthesis of derivates like Bis-GMA, and thus only small amounts are leachable due to possible contaminations from the use of BPA derivatives [38,44]. Even though BPA is at the center of current research, cytotoxic, genotoxic, proinflammatory, and even mutagenic effects have been shown for various compounds used in dental resins [45,46,47,48,49,50,51,52]. Considering the advancements and changes in the composition of resin composites [53], monomers, as well as further compounds, e.g., additives eluting from dental resins, should be investigated. The biocompatibility of dental materials may be evaluated by using various in vivo or in vitro techniques [54] and the collection of reliable data depends on the application of adequate detection and quantification methods [55]. As a result of varying clinical situations occurring in vivo, these studies show a large spread width of released monomer concentrations [56,57,58,59]. Therefore, it has been established in biocompatibility testing to verify the results of standardized in vitro studies by in vivo trials [60]. The general setup of in vitro studies on the leachability of monomers from dental resins consists of an experimental part to produce an eluate by incubating resin samples in an extraction medium, and an analytical part to identify and quantify monomers within the eluate. However, as of today, comparative analysis of current in vitro studies is limited due to heterogeneous sample design as well as the diverse application of analytical methods with various extraction media and not standardized observation periods [34]. However, without a systematic meta-analysis, the impact of monomer release from dental resins on patient health remains unclear. The present review aims to provide information on each step of the experimental and analytical setup regarding the in vitro identification and quantification of eluting compounds from dental resins to develop a basis for future meta-analytical evaluations.

## 2. Sample Design

In current literature, samples are commonly disc-shaped and of various sizes, while the removal of the oxygen inhibition layer is either not performed or not mentioned [17,61,62,63,64,65]. However, the surface area and the oxygen inhibition layer influence the amount of eluting monomers heavily, and therefore sample design is a limiting factor in current research. ISO 4049 specifies the requirements for dental polymer-based restorative materials, and many authors recommend complying with ISO 10993-12 for the sample design [62,66,67,68,69]. ISO 10993-12 regulates sample preparation and reference materials for the biological evaluation of medical devices and recommends regular-shaped samples with a defined surface area [70]. Additionally, ISO 10993-12 specifies in dependency of the surface area the corresponding solvent volume and as a consequence the extraction ratio (Table 1) [70]. To our knowledge, there are no studies on the effects of different extraction ratios on the release of specific monomers, but it is known that the extraction ratio affects the amount of released monomers from the polymer matrix strongly [34,71,72]. The lack of studies with a uniform extraction ratio hinders meta-analytical analysis and limits the comparison to common restoration sizes [34]. Therefore, future studies should comply with ISO 10993-12.

Besides the extraction ratio, the oxygen inhibition layer needs to be considered when evaluating the release of monomers from the polymer network. The oxygen inhibition layer contains unpolymerized monomers [73,74], which can be eluted, and especially TEGDMA concentrations seem to be elevated [71]. Therefore, the removal of the oxygen inhibition layer leads to fewer eluted monomers as well as reduced cytotoxicity, and thus the removal of the oxygen inhibition layer or prevention of its formation is recommended in clinical practice [75,76]. However, in many studies on the leachability from dental resins, the oxygen inhibition layer was either not or ineffectively removed [61,62,63,64,65]. Recent literature shows that water-spray or ethanol treatments are ineffective methods to remove the oxygen inhibition layer [77]. For research purposes, nitrogen [78,79,80], argon [81,82], or carbon dioxide [83] atmospheres have been used to produce samples without an oxygen inhibition layer. Even though these methods are effective, they are costly and not applicable in clinical practice. Effective methods that are applicable in vitro and in vivo include methods that prevent oxygen contact, e.g., glycerin gel or mylar strips, and mechanical methods, e.g., specimen polishing with a defined removal of 0.2 mm [84,85,86]. Recent studies show that these methods are well suited for the in vitro investigation of the release of monomers from dental resins [35,66,87,88,89]. Consequently, the removal or prevention of the oxygen inhibition layer should be included in the sample preparation process and based on the clinical workflow. Besides the oxygen inhibition layer, the surface roughness may influence monomer and BPA elution, but to our knowledge, there are no studies on the effect of surface roughness on the monomer release. Therefore, we recommend polishing procedures corresponding to the standard clinical workflow.

## 3. Selection of the Extraction Medium

Various solvents, such as water, ethanol-water mixtures, methanol, acetonitrile, tetrahydrofuran, cell culture media, artificial saliva, and collected saliva, have been used as extraction media in studies investigating the in vitro release of monomers from the polymer matrix [9,17,61,64,90,91,92,93]. However, interactions with the extraction medium due to substance-specific properties, such as molecular size and other chemical characteristics, significantly alter elution [94]. Literature assumes that in vivo conditions are somewhere between the less aggressive water solvent and the more potent ethanol solvent [65]. Hence, the US Food and Drug Administration (FDA) recommends a 75 vol. % ethanol/water solution which is supposed to be a good food simulator (alcoholic beverages, fruits, and syrup) and therefore clinically relevant [9]. Due to this recommendation, many studies investigating the leachability of monomers from dental resins used a 75% vol. ethanol/water solution [9,33,95,96,97,98]. However, the solubility parameter of ethanol and Bis-GMA is almost equal, which leads to the softening of resins, with maximum softening reached at 75% vol. ethanol/water [99,100,101]. Ethanol/water solutions penetrate the polymer matrix, especially of Bis-GMA-based resins, and degenerate it irreversibly by expanding the space around the polymers and creating soluble units [94,101]. Considering these findings, it is questionable whether using a 75 vol. % ethanol/water solution results in clinically relevant data [71]. Supporting this hypothesis, many studies found significantly elevated Bis-GMA, TEGDMA, and UDMA levels in 75% vol. ethanol/water solutions compared to artificial saliva [36,72,102,103]. BPA was only detected in samples immersed in a 75% vol. ethanol/water solution [72,102,103]. In addition to elevated monomer concentrations, monomer elution is prolonged in 75 vol. % ethanol/water solutions [35]. Considering these findings, 75 vol. % ethanol/water solutions cannot be recommended to simulate the intraoral environment for the investigation of the leachability of dental monomers. Similarly, immersion in methanol leads to an increased monomer release compared to water or artificial saliva and should be therefore also avoided [92]. Besides alcohol-based solutions, cell culture media were used as extraction solvents, but it was shown that they may lead to false-negative results especially regarding TEGDMA detection due to the binding of albumin to it [17]. Instead, water, artificial saliva, or human saliva should be favored as extraction media. Literature shows similar concentrations of released compounds when comparing distilled water to artificial saliva [17,103,104]. In aqueous environments, mainly hydrophilic molecules of small sizes, such as TEGDMA, elute, while long-chain hydrophobic molecules, such as BisGMA, are hardly released [105,106]. Comparing the eluates from samples incubated in distilled water, artificial saliva, and collected salvia, the latter contains lower monomer levels, as proteins contained in collected salvia bind eluting monomers [104]. When using collected human saliva, the probands must not have restorations [107] and blank samples for the analytical procedure are needed to avoid false positives [91]. In conclusion, the most comparable results can be achieved with collected saliva, but blank samples and a robust analytical procedure are required. Moreover, water and artificial saliva are an option, but in contrast to former assumptions, they may even present slightly increased concentrations of eluted monomers. Further studies on the impact of the composition of human saliva on the elution from dental resins and the effect of protein-bound monomers on the metabolism are necessary.

## 4. Incubation Parameters

The amount of eluted compounds highly depends on incubation parameters, such as the incubation time, buffering systems, solvent, monomer saturation, and pH value [71,108,109,110]. Under extreme temperature (100 °C) and an alkaline (pH 13) or acid (pH 1) environment, BPA is released due to hydrolytic degradation of Bis-GMA or bisphenol A diglycidylether (BADGE) [111]. Particularly very alkaline conditions seem to promote BPA elution [25]. A long-term study on the leakage of composite resins found that the effect of pH varied among monomers: more BPA was eluted at pH 8 than at pH 4 and 6, while the elution of TEGDMA followed the opposite trend [112]. Considering these findings, future studies on the elution from dental resins should report incubation parameters, especially the pH and temperature of incubation, whereas incubation at 37 °C is recommended to simulate in vivo conditions [34].

In some studies, samples are incubated after a post-irradiation cure, usually 24 h in the dark [61,113,114,115]. Incubation after the post-irradiation cure leads to lower concentrations of released substances [105]. Since direct incubation corresponds to the clinical workflow, it is recommended [114,116].

Studies on the long-term release from dental resins need to take salivation of the oral environment into account and must refresh the extraction medium to avoid saturation, which might lead to the underrepresentation of in vivo conditions [97,117]. This solvent refresh is usually performed once per week [33,35,94]. Furthermore, the stability of dental monomers in water, artificial salvia, or collected saliva must be considered, especially in long-term studies. Presumably, passive hydrolysis reactions lead to the degradation of monomers in water [118]. As a consequence, many studies showed decreasing monomer concentrations when successive incubation periods were analyzed [17,61,103,119,120]. This was not observed or observed to a lesser extent in other extraction media, such as ethanol/water mixtures or lactic acid [103,119]. Passive and/or enzyme-catalyzed hydrolysis, such as in collected saliva, cleaves the ester bonds of the methacrylate groups of BisGMA, TEGDMA, and UDMA [52,121,122,123]. Initial decreases in concentrations were observed after only six hours of incubation [103]. Due to the incomplete hydrolysis of dental monomers, molecules with different numbers of cleaved methacrylate groups may be present simultaneously [122,124]. These hydrolysis products each have different chemical properties as well as molecular masses, and thus detection requires adjustments to the analytical method [124,125]. In conclusion, the analysis of long incubation intervals could lead to the underrepresentation of the in vivo monomer release. Therefore, degradation products should be measured additionally [124,126,127]. Regular refreshment of the extraction medium and the cumulative determination of monomer concentrations are recommended. Cumulative analysis has already been performed in several studies [35,102,128].

In summary, the objective of in vitro incubation is the simulation of in vivo conditions. Therefore, incubation parameters should be based on the intraoral environment, the extraction medium should be refreshed regularly, and samples should be incubated directly. For future meta-analytical evaluation and comparability between studies, a 24-h incubation period should be included in every study on the elution from dental resins [34].

## 5. Analytical Setup

A wide range of techniques has been used to detect and quantify substances eluting from dental resins [118]. Many older methods such as infrared spectroscopy are nowadays regarded as outdated since the signals are not molecule-specific, the interpretation of spectra is difficult, and quantification unreliable [34]. Nowadays, the analytical setup consists of the separation of the eluate by chromatography followed by subsequent detection of the eluted compounds by optical methods or mass spectrometry. The FDA and recent literature recommend high-performance liquid chromatography (HPLC) and gas chromatography (GC) as separation methods [34,66,129]. Despite the recommendation of GC [66,129] and its application in various studies [107,109,112,130], recent literature showed that the high operating temperatures of GC lead to the overestimation of the leakage of BPA due to thermal degradation of Bis-GMA [64]. Bannach et al. [131] investigated the thermal stability of Bis-GMA, ethoxylated bisphenol A dimethacrylate (Bis-EMA), UDMA, and TEGDMA and found that thermal decomposition starts between 178 and 297 °C, which corresponds to the temperatures between 280 and 400 °C occurring during GC [109,130]. Consequently, GC is not capable of detecting Bis-GMA, Bis-EMA, or UDMA, but only corresponding thermal degradation products [56,132,133,134]. Due to the thermal degradation of BPA derivatives, BPA was found by GC in all samples regardless of the solvent, but HPLC-MS detected BPA only in samples immersed in methanol [64]. HEMA is a potential degradation product of UDMA, and therefore detection and differentiation between them are hindered [109]. Additionally, GC analysis of analytes from aqueous samples requires time-consuming sample preparation [135]. In conclusion, GC should be avoided for the separation of monomers with a high molecular weight in eluates from dental resins due to their thermal instability [136]. Instead, HPLC is the recommended separation method for the analysis of eluting monomers from dental resins. However, GC can be applied for low molecular weight, volatile, and thermally stable substances, e.g., additives contained in dental resins [133,136], as it allows accurate quantitative determination within complex mixtures, including trace amounts of compounds down to parts per trillion in some cases [137].

Most light absorption spectroscopy (UV/Vis) and mass spectrometry (MS) detectors coupled with HPLC are suitable for the identification and quantification of compounds in the eluate [138]. UV/Vis detectors are available in many analytical laboratories due to their easy use, low cost, and near-universal field of application [138]. It is known from other scientific fields that the sole use of UV/Vis can lead to the overestimation of analyte concentrations due to the presence of coeluting substances [139,140,141]. Hope et al. found that the wrong identification of a coeluting substance, probably a photoinitiator, in eluates from an experimental dental resin led to the overestimation of BPA levels by 30-fold when comparing UV/Vis to MS detection [66]. This discrepancy between UV/Vis and MS detection was also found in a recent study on the monomer elution from temporary crown and bridge materials [120]. Therefore, the sole identification and quantification by UV/Vis are not recommended and more sensitive and specific methods like MS should be used. MS is a very sensitive and selective method for the detection of unknown substances and degradation products in eluates from cured as well as uncured resins [67]. In this context, electrospray ionization (ESI), atmospheric pressure chemical ionization (APCI), and atmospheric pressure photoionization (APPI) are available for the ionization of nonvolatile and thermally unstable analytes [142]. Advanced MS detectors coupled with HPLC (HPLC-MS) allow tandem mass spectrometric analysis (HPLC-MS/MS), which increases the specificity of the analysis by the fragmentation of a pre-selected ion and specific detection of selected fragmentation products [143]. In recent years, high-resolution mass spectrometry (HRMS) following HPLC separation has proven to be a viable alternative due to high mass accuracy and sensitivity even in full-scan mode [144]. Even more information about the molecular structure can be obtained by the combination of HRMS and MS fragmentation [145]. Accordingly, HPLC coupled with MS, preferably HRMS and/or tandem MS, is the recommended analytical method for the analysis of monomers eluting from dental resins.

## 6. Detection/Qualitative Analysis

**Figure 1:**
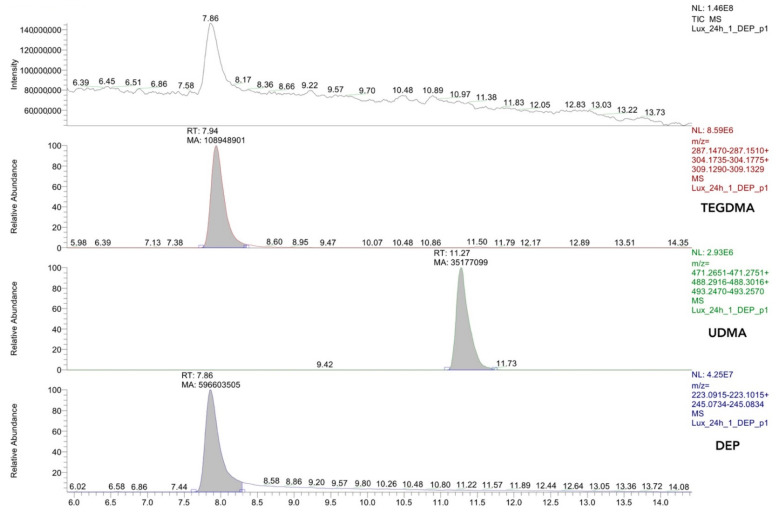
HPLC-MS Chromatogram of a Luxatemp Automix Plus (DMG Chemisch-Pharmazeutische Fabrik, Hamburg, Germany) sample immersed in HPLC grade water with diethyl phthalate (DEP) as an internal standard. At the top, the chromatogram and then, from top to bottom, the extracted ion chromatograms of TEGDMA, UDMA, and the internal standard DEP, respectively. Definitive peak identification is accomplished by the relative abundance of the corresponding molecular mass. This chromatogram was prepared for a study on the monomer elution from resin-based temporary crown and bridge materials [120].

**Figure 2:**
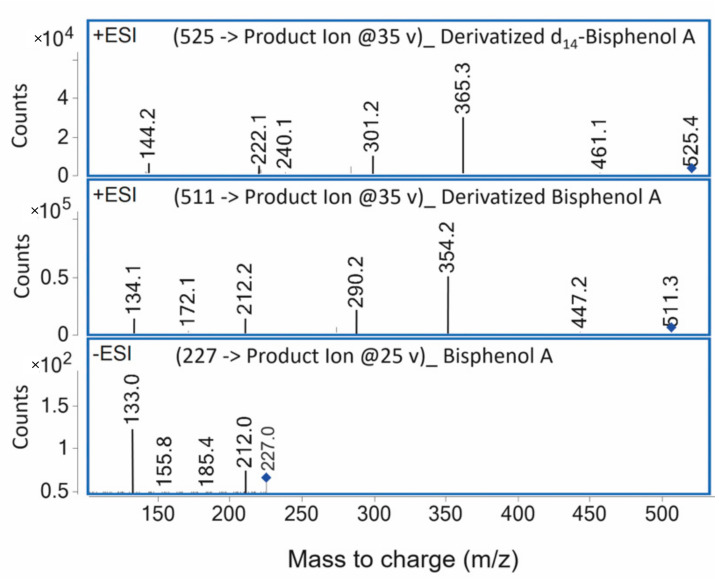
Tandem mass spectrometry fragmentation spectra of derivatized isotope-labeled BPA (**top**), derivatized unlabeled BPA (**middle**), and underivatized BPA (**bottom**). The spectra were obtained by HPLC-MS/MS with negative ionization for native BPA and positive ionization for BPA derivatized with pyridine-3-sulfonyl chloride. The unique and most abundant product ion is highlighted by a blue square. This mass spectrum was prepared for a study on the monomer elution from resin-based temporary crown and bridge materials [120].

An essential part of a reliable study design for the detection of monomers is a clear definition of the analyte and the estimation of the limit of detection (LOD), which should be as low as technically achievable. In a typical HPLC-MS analysis with electrospray ionization, the retention time together with the mass of the molecular ion and in tandem MS as well as the fragmentation spectrum of the molecular ion are used to identify the analyte (Figure 1 and Figure 2).

The fragmentation spectrum of a tandem MS analysis contains fragmentation ions, each of which has a separate peak in the mass spectrum [146]. The most abundant, unique fragmentation ion of an analyte is used for quantification (quantifier ion), and less abundant, unique ions are used for detection (qualifier ions) [147,148,149]. By analyzing more than one qualifier ion, confidence in detection can be increased [150]. For the analysis of unknown compounds, mass spectrometry allows the use of libraries with mass spectra collected from literature, like the NIST mass spectral library (National Institute of Science and Technology, Gaithersburg, MD, USA), which contains among other substances dental compounds [56,151,152]. Commercially available and open-source libraries help to identify unknown substances [153]. However, these libraries mostly contain spectra from GC-MS obtained after ionization with “hard” ionization techniques [154]. Due to the lower reproducibility of retention properties in HPLC instruments, the availability of libraries with spectra obtained by HPLC-MS with soft ionization techniques is limited [154,155,156]. Therefore, older studies on the release of dental monomers using libraries, e.g., the NIST applied GC-MS analysis [56,151,152]. However, the identification of an unknown compound should not solely rely on the comparison between a library and an experimental mass spectrum because co-eluting compounds may compromise the fragmentation spectra of the analyte, and library spectra do not reflect experimental conditions [144]. Therefore, the regulation 2002/657/EC issued by the European Union recommends the use of calibration solutions and an internal standard to validate the qualitative analysis [157]. For a clear definition of the reference substances, it is best practice to report their molecular masses and unique Chemical Abstract Service Registry Numbers (CAS Registry Number). This is especially important in dental research, as recent research shows that the trivial name UDMA is used for a variety of molecules, and Bis-EMA is used for molecules of different degrees of ethoxylation [55,130].

Furthermore, the LOD is essential for the assessment of the reliability of detection because compounds present at concentrations below the LOD cannot be detected though may be released and have a toxicological impact [158]. According to the International Union of the Pure and Applied Chemistry (IUPAC), LOD is defined as the lowest reliably detectable concentration of an analyte [159]. Various methods for determining the LOD can be found in the literature. The most common approach is the calculation based on the signal-to-noise ratio, but an experimental determination by a dilution series is also possible [160]. If the analyte is available, the Joint Research Centre of the European Commission recommends using 3.9 times the quotient of the standard deviation of the blank (pseudo-blank) signals and the slope of the calibration curve for the determination of a reliable LOD [161]. Therefore, LOD can be improved either by the reduction of noise or by the increase of signal strength [162]. Common ways to reduce noise are sample clean-up, temperature control of the column, and purity of the reagents and solvents [162]. Consequently, lower LODs are achievable with HPLC grade extraction media than with other media such as collected saliva. The signal strength can be increased by the injection of larger quantities of the sample, a more sensitive detector, and the choice of the mobile phase and column to change peak width [162]. Another way to improve the LOD might be derivatization. Recently, it was shown that the derivatization of BPA in composite eluates allows mass spectrometric detection in the more sensitive positive ESI mode and therefore leads to lower LODs [120,163].

As most studies on the leachability from dental resins do not report the LOD, only the positive and not the negative results can be interpreted [34]. The LODs in the current literature for Bis-GMA range from 0.07 µg/mL to 1.18 μg/mL [86,89,164,165] and for BPA from 0.003 µg/mL to 0.075 µg/mL [66,165,166]. The LODs for UDMA are reported between 0.075 μg/mL and 0.63 µg/mL [86,89,164,165]. Respective values for TEGDMA vary between 0.022 μg/mL and 0.808 µg/mL [86,89,164,165,167] and for HEMA between 0.022 μg/mL and 2.43 μg/mL [86,87,164,165,167].

## 7. Quantitative Analysis

**Figure 3:**
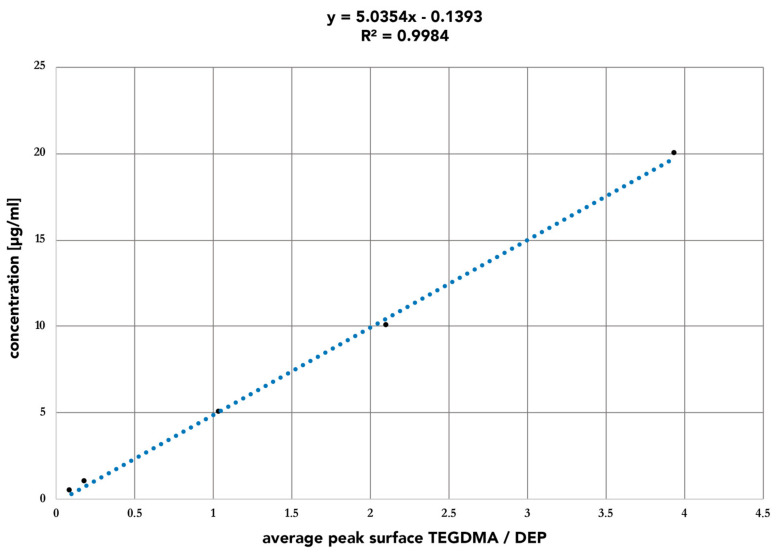
Calibration curve of TEGDMA in relation to the internal standard diethyl phthalate (DEP) including the plot of residuals. The data are fitted by a linear regression model and assessed by the determination coefficient r2. This calibration curve was prepared for a study on the monomer elution from resin-based temporary crown and bridge materials [120].

For reliable quantitative analysis, precise calibration is vital. Calibration methods reconstruct the dependence between the analytical signal and the concentration of internal and/or external standards, which correspond to the relationship between the signal and the concentration of the analyte in the sample [168,169]. This relation is used to prepare a calibration curve and the data are fitted by a mathematical function, which usually is linear regression [170]. Calibration can be performed using single-point, double-point, or multi-point calibration, whereas today only multi-point calibration is considered acceptable [169]. For multi-point calibration, 5–10 concentrations of each standard in the range of 0–150% or 50–150% of the concentration likely to be encountered are analyzed in duplicates or triplicates [171,172,173]. Depending on the expected eluted concentrations, most studies analyzed a series of uniformly distributed standard solutions with concentrations between 0.005 ng/mL and 1000 µg/mL [64,89,107,120,164,171,172,173,174,175,176]. When linear regression is used, the linearity of the calibration curve is often assessed by the correlation coefficient r or the determination coefficient r^2^ [177]. The latest guideline by the clinical and laboratory standards institute (CLSI) considers a correlation coefficient r ≥ 0.975 or r^2^ ≥ 0.95 as sufficient evidence for linearity [178]. However, it was shown that even in some cases with r > 0.99 linearity is not always fulfilled and thereby a plot of residuals and possibly a lack of fit or Mandel’s fitting test can be performed to verify a normal distribution of calibration points around the line, which is expected in cases of a true linear fit [172,179,180]. Any curvature of this plot is an indication of a lack of fit and therefore suggests the need for a non-linear regression model [179]. However, linear calibration is preferred over non-linear calibration models because of easy calculation and statistical assessment [180]. The lowest calibration standard used is considered the limit of quantification (LOQ) and the signal corresponding to this calibration standard should be at least five times higher than the blank signal [181]. In order to evaluate the strength of the study, both the LOQ and the calibration curve, including its plot of residuals, the correlation or determination coefficient, and the slope of the curve, should be reported (Figure 3).

The LOQs in the current literature for Bis-GMA range from 0.01 µg/mL to 3.51 μg/mL [35,86,89,164] and for BPA from 0.00003 µg/mL to 0.2 µg/mL [64,120,182,183,184]. The LOQs for UDMA are reported between 0.005 μg/mL and 1.90 µg/mL [35,86,89,164]. Respective values for TEGDMA vary between 0.005 μg/mL and 2.424 µg/mL [35,86,89,164,167] and for HEMA between 0.2 μg/mL and 7.36 μg/mL [35,86,164,167].

## 8. Calibration Techniques

The most common calibration technique is known as external calibration and consists of the separate preparation and analysis of standards and samples [185]. External calibration is prone to matrix effects and does not consider losses in sample preparation or analysis [185,186]. Therefore, ISO Guide 33:2015 recommends using this technique only for matrix-free samples [187]. This source of error is reduced by calibration methods that use standards present in the sample during preparation and analysis [188]. These methods are known as internal calibration and standard addition calibration [186]. For internal calibration, a constant amount of an internal standard similar to the analyte of interest is added to the samples and calibration standards to obtain a calibration factor that is applied to the analyte signal (Figure 1) [185,186]. Most commonly, a set of standards containing the range of expected concentrations of analyte and a single concentration of internal standard is analyzed to obtain the corresponding calibration curve (Figure 3) [189]. Internal calibration requires substances with similar retention times—ideally, isotope-labeled standards of the analyte—and multiple standards may be required when analyzing multiple analytes [190]. Using standard addition, the signal change by adding increasing amounts of a standard to aliquots of the sample is measured and thereby the original concentration is calculated by applying a linear function fitting all experimental points [191]. Consequently, standard addition leads to long-lasting calibration procedures because the individual calibration of each sample is required [168]. However, in contrast to other methods, this is the only technique not affected by systematic matrix errors and is recommended for complex matrices [185].

**Figure 4:**
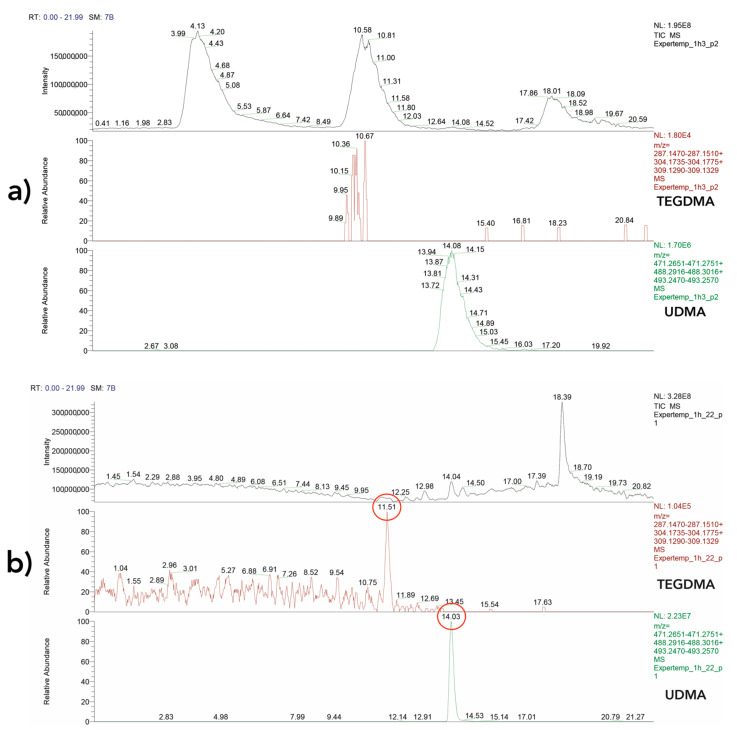
(**a**) HPLC-MS Chromatogram of an ExperTemp sample (Ultradent, South Jordan, USA) immersed in HPLC grade water and Gibco Antibiotic-Antimycotic solution. Due to the overlapping peaks of the antibiotic-antimycotic solution, the peaks corresponding to the masses of TEGDMA and UDMA are not identifiable. (**b**) Chromatogram of an ExperTemp sample (Ultradent, South Jordan, USA) immersed in pure HPLC grade water. The peaks corresponding to TEGDMA and UDMA are highlighted. This figure was taken from preliminary tests made by our working group for a study on the elution of monomers from resin-based temporary crown and bridge materials [120].

Most studies on the release of residual monomers from dental resins are limited by the inaccuracy of external calibration [107]. However, the use of internal standards, especially in complex matrices, such as urine and artificial or collected saliva, is recommended [190]. In the literature, caffeine [64,134,192], diethyl phthalate [109,193,194], or standards labeled with stable isotopes [57,62,66,163] have been used as internal standards. Due to its omnipresence, caffeine should not be used in studies using collected human saliva as the solvent [107]. Special caution is required when using diethyl phthalate, as diethyl phthalate is used in dental materials and has already been detected in different composites [192,195]. However, the internal standard must not be present in the sample. Otherwise, quantification will be falsified [196]. In order to avoid this, deuterated diethyl phthalate has been used in more recent studies [197,198]. When adding internal standards or other compounds to the sample, it is important to rule out the introduction of matrix effects by coeluting substances because they may lead to decreased accuracy and sensitivity [199]. Recent studies added an antibiotic–antimycotic mixture to the solvent to avoid microbial colonialization [61,63], but a recent trial for a study of our working group showed that the peaks corresponding to the mixture overlapped the peaks of relevant dental monomers heavily and may have introduced matrix effects to the analytical procedure (Figure 4).

For this reason, every added compound needs to be evaluated for the introduction of matrix effects. Because of similar properties to the analyte and close elution to the analyte, while being well separable, analytical standards labeled with stable isotopes, such as deuterium or ^13^C, are the most appropriate internal standards [190]. Future studies should use internal standards, preferably labeled with stable isotopes, for calibration.

## 9. Method Validation

Method validation is mandatory before routine analysis for all analytical methods. International guidelines, by the FDA [177], European Medicines Agency (EMA) [181], and the International Union of the Pure and Applied Chemistry (IUPAC) [200] provide information on the validation of analytical methods. According to these guidelines, the main parameters which need to be validated are linearity, accuracy, precision, specificity, selectivity, matrix effects, and stability [201]. Key parameters of validation, especially the limits of detection (LOD) and quantification (LOQ) [34,66] as well as the slope of the calibration curve [201], should be stated in the method validation section, so readers can evaluate the strength of the study. Very few studies on the elution from dental resins reported these validation parameters [107,163,174].

## 10. Conclusions

Due to the diverse application of dental resins in various areas of dentistry, it is necessary to develop reliable, evidence-based analytical methods for the detection and quantification of eluting compounds. Analytical and experimental methods used in recent literature vary widely and validation parameters are reported rarely. We propose a consistent study design to collect reliable data that allows consistent meta-analytical evaluation. When researching the in vitro elution from dental resins, the following criteria should be met:

1. The surface area of the sample and the corresponding solvent volume should be standardized according to ISO 10993-12 and following the clinical workflow, the oxygen inhibition layer of the samples should be removed.
2. In order to achieve results comparable to in vivo conditions, solvents, such as water, artificial saliva, or preferably collected saliva, should be used.
3. Incubation parameters should mimic in vivo conditions. Therefore, immediate incubation at 37 °C and a frequent solvent refresh is recommended. For later meta-analysis, a 24-h incubation period should be included in all studies.
4. HPLC-MS, preferably with HRMS and/or tandem mass spectrometry, calibrated by internal standards is the recommended analytical method for detection and quantification.
5. CAS Registry numbers and molecular weights of standards and detected substances must be reported.
6. The analytical method should be validated properly. Key validation parameters, e.g., the LOD, LOQ, and the calibration curve, including its interception, slope, and the plot of residuals, need to be reported for interpretation of study results.

## Figures and Tables

**Table 1:** Recommended extraction ratios according to ISO 10993-12.

Thickness (mm)	Extraction ratio ± 10%
≤0.5	6 cm^2^/mL
>0.5	3 cm^2^/mL
Irregular shaped sample	0.1–0.2 g/mL, 6 cm^2^/mL

## Data Availability

Not applicable.

## Abbreviations

Many different abbreviations are used in the scientific literature on the leachability of compounds from the polymer matrix of dental resins. This section provides an overview of the most commonly used abbreviations:

APCI	Atmospheric pressure chemical ionization
APPI	Atmospheric Pressure Photoionisation
BADGE	Bisphenol A diglycidyl ether
Bis-EMA	Ethoxylated bisphenol A dimethacrylate
Bis-GMA	Bisphenol A diglycidyl methacrylate
Bis-HPPP	Bis-hydroxy-propoxy-phenyl-propane
BPA	Bisphenol A
CAS	Chemical Abstracts Service
ESI	Electrospray ionization
GC	Gas chromatography
HEMA	2-hydroxylethyl methacrylate
HPLC	High-performance liquid chromatography
HRMS	High-resolution mass spectrometry
LC	Liquid chromatography
LOD	Limit of detection
LOQ	Limit of quantification
MS	Mass spectrometry
PDA	Photodiode array,
TEGDMA	Triethylene glycol dimethacrylate
UDMA	Urethane dimethacrylate
UV/Vis	Ultraviolet/visible
